# Extracted metabolite from *Streptomyces Levis* ABRIINW111 altered the gene expression in colon cancer 

**Published:** 2018

**Authors:** Parisa Fatourachi, Behnaz Faramarziyan Azimi Maragheh, Seyede Momeneh Mohammadi, Behnaz Valipour, Alireza Dehnad, Hojjatollah Nozad Charoudeh

**Affiliations:** 1 *Stem Cell Research Center (SCRC), Tabriz University of Medical Science, Tabriz, Iran*; 2 *Payam Noor University, Tehran, Iran*; 3 *Higher education institute of Rab-Rashid, Tabriz, Iran*; 4 *Biotechnology Department, East Azerbaijan Agricultural Education Center, AREEO, Tabriz, Iran *

**Keywords:** Colon cancer, Metabolites, oncogenes, *Streptomyces Levis*, ABRIINW111

## Abstract

**Aim:**

In this study we attempt to indicate anti-carcinogenic influence of ether extracted metabolites of *Streptomyces Levis* sp. on gene expression in colon cancer.

**Background:**

Colon cancer is one of the most prevalent cancers worldwide. In recent decades, researchers have been seeking the treatment for cancer. Natural products are valuable compounds with fewer side effects in comparison to chemotherapy drugs.

**Methods:**

Secondary metabolites were extracted with the inoculation of bacterial sample in Mueller Hinton Broth. MTT assay was done to evaluate the cytotoxicity effect of metabolites on SW480 cells. qRT-PCR was performed to observe effects of metabolites on Bcl-2, P53, SOX2, KLF4, β-Catenin, SMAD4, K-ras, BRAF genes expression in colon cancer.

**Results:**

The metabolites exhibited cytotoxic effects on colon cancer in a dose/time dependent manner (P < 0.001). After 48 h treatment, fold expression of Bcl-2, SOX2, β-catenin, K-ras, BRAF genes fold of expression were decreased, whereas P53, KLF4, SMAD4 genes were increased in treated cells (P < 0.001).

**Conclusion:**

These findings indicate that ether extracted metabolites of *Streptomyces Levis* ABRIINW111 have anti-carcinogenic effects on colon cancer.

## Introduction

 Colon cancer is a global challenge worldwide (1-3). Genetic and epigenetic alterations in the normal colonic epithelium lead to colon adenocarcinoma (4). Benign adenomatous polyp is the first step, then polyps develop into a malignant adenoma with high-grade dysplasia which subsequently transform into invasive cancer (5). Changes in genes expression is a significant prognosis factor for initiation and progression of colon cancer which could be a biomarker for targeting colon cancer. Colon cancer is most commonly initiated by changes in the Wingless/Wnt signaling pathway. Inactivation of tumor suppressor genes and activation of oncogenes such as Bcl-2, P53, SMAD4, BRAF, K-ras, Beta-catenin, SOX2, and Klf4 lead to development of colon cancer. Some of the predominant alterations that have been demonstrated to play an important role in initiation of colon cancer include K-ras, P53; TGFBR2 and SMAD4 as elements of the TGF-p signaling pathway are involved too. 

Bcl-2 family control the integrity of mitochondrial membrane. The anti-apoptotic proteins including Bcl-2, BAG, Bcl-x, Bcl-XS, Bcl-XL, Bcl-w and the pro-apoptotic proteins like Bax, Bid, Bak, Bad, NOXA, and PUMA are members of this family (6-10). Bcl-2 acts as an anti-apoptotic member and controls the apoptosis by several mechanisms such as releasing of ions into the cytoplasm through altering the permeability of the intracellular membranes (11, 12). P53 as a transcription factor has a suppressor activity and it is mutated in 50% of primary colon cancers (13). Expression of several pro-apoptotic genes such as Bax, NOXA, and PUMA is controlled by P53 (14-16).

Sox2 is a member of the Sox gene family, belongs to the SOX B1 subgroup. It acts to preserve development potential and encode transcription factors with a single HMG DNA-binding domain (17). The Kruppel-like factor (KLF) family of genes regulates a wide range of cellular processes such as differentiation, migration, apoptosis, proliferation, tumor formation and inflammation. KLF4 is exceeding and has observed in the gastrointestinal epithelial cells, skin, and endothelial cells in vascular system (18-21). KLF4 as a regulator of cell proliferation, induces cell cycle arrest at G1 to S phase in a p53-dependent manner by activation of p21WAF/Cip1 gene as the negative cell-cycle-regulatory cyclin-dependent kinase inhibitor (22, 23).

ß-catenin is one of the elements of the APC/ß-catenin/TCF/Lef pathway and its expression is increased by activation of the Wnt signaling pathway. It plays a main role in cancers such as melanoma, and gastric cancer (24-26).

SMAD4, mutated mostly in colon cancers, belongs to the SMAD family of genes and acts as a tumor suppressor gene. In the transforming growth factor-p (TGF-p) signaling pathway, SMAD4 codes cytoplasmic mediators (27, 28).

K-ras is one of the important elements in the Ras/MAPK signaling pathway. This signaling pathway, by inducing the synthesis of cyclin D1, plays a key role in apoptosis, differentiation and cell proliferation. Mutation of the K-ras as a proto-oncogene activates this pathway, which is found in 36% of colorectal cancers (29-33). Three RAF genes that are regulated by binding to RAS, mediate the RAS-induced cellular response to growth signals by encoding cytoplasmic serine–threonine kinases. BRAF is one of the three known RAF genes that have resulted from gene duplication (30).

The SW480 cell line is obtained from the colon adenocarcinoma with moderate level of differentiation. Previous studies have illustrated that SW480 cell line displays most of the genetic changes which are seen in aggressive colon cancers, including a K-ras mutation (34), p53 mutation (35), loss of the DCC gene on chromosome 18 (36). 

*Streptomyces sp* as the largest genus among actinomycets, produces a wide range of important secondary metabolites, including antimicrobial and anticancer (37). For example, Rapamycin – isolated from the soil bacteria *Streptomyces hygroscopicus* - has revealed anticancer activity (38-40). Recent studies are focused on microbial natural products as the most promising source for developing better antibiotics (41). In our screening program for producing bioactive compounds, the diethyl ether extracted from *Streptomyces Levis ABRIINW111* has shown strong activity against colon cancer cells (unpublished data). 

One of best methods for cancer therapy is using natural products. They act as anti-cancer agents without serious side effects. They can induce apoptosis and change genes expression in cancer cells (42). Because of these advantages, metabolites as natural products can be a good choice for cancer therapy. In this study we evaluated the *Streptomyces Levis ABRIINW111 *metabolites effect on the pro-apoptic, anti-apoptotic and several oncogenes to understand how these metabolites could be effective products in cancer therapy. 

## Methods

*Streptomyces Levis ABRIINW111* was purchased from the Department of Microbial Biotechnology, AREEO, Tabriz, Iran. Metabolites were extracted as described, bacteria was cultured in Nutrient agar medium (Sigma /70148) at 29 ^°^C for 7 days. loop full of bacteria was inoculated into 25 ml of Mueller Hinton Broth (Sigma /70192) and incubated while agitating on shaker incubator set at 70 rpm at 29 ^°^C for 36 h (43). As previously described, we used spectrophotometrical reading and chose turbidity 620 nm, 0.08 O.D, as an appropriate concentration for inoculation(43). After fermentation time, 1 ml of pre-culture was used to inoculate 1,000-ml Erlenmeyer flasks; each contained 150 ml of fresh Mueller Hinton Broth medium. The fermentation was carried out at 29 ^°^C for 7 days on shaker incubator set at 70 rpm, centrifuged at 4000 rpm for 20 minutes. The Cell free filtrate was mixed with equal volume of Diethyl ether (1:1 V/V) shaken for 1 h at 175 rpm, extracted by Diethyl ether (100921/ Merck), using separating funnel. Finally, the obtained organic extract was concentrated at room temperature until 0.01 gr reddish brown crude extract obtained; the resulting extract was kept at 44 °C until used (43). Also, *Streptomyces Levis ABRIINW111* metabolites fractions were analyzed by HPLC method (44). Metabolites were dissolved in final concentrations of 100, 500, 1000, 2000, 5000 ng/ml in DMSO (43, 45).

**Cell culture and MTT assay**

SW480, a human colon cancer cell line, was obtained from Pasteur Institute (Tehran, Iran). Cells were cultured in RPMI 1640 medium supplemented with 10% FBS, 1% penicillin and streptomycin in 5% CO_2_ at 37 ˚C^˙^. 

For MTT assay, 1×10^4^ SW40 cells were seeded per well in 96-well micro plates with 100μl of culture medium containing RPMI 1640 medium supplemented with 10% FBS, 1% penicillin and streptomycin in 5% CO_2_ at 37 ˚C and incubated for 24 h. Metabolites were diluted in culture medium with less than 0/1% DMSO (Dimethyl Solfoxide) and various concentrations of bacterial metabolites (100, 500, 1000, 2000 and 5000 ng/ml) were incubated in 5% CO_2 _at 37 ˚C for 24, 48 and 72 h. Untreated cells served as control. After incubation time, supernatant was carefully replaced with 20 µL of MTT reagent (M6494/ Sigma) {3-(4,5-dimethyl thiazol-2-yl)-2,5-diphenyl tetrazolium bromide (5mg/ml), incubated in 5% CO_2_ at 37 °C for 4 h and 100 µL of DMSO was subsequently added to dissolve the appeared colored formazan crystals. The optical density was measured at 570 nm with reference wavelength 630 nm by micro plate Elisa reader (Biotek ELx 808, USA). 

**Real time PCR**

The 1×10^6^ cells were cultured in RPMI 1640 medium supplemented with 10% FBS, 1% penicillin and streptomycin in 5% CO_2_ at 37 ˚C. After 24 h, supernatant was removed and cells were treated in 1000 ng/ml of metabolites and incubated in 5% CO_2_ at 37 °C for 48 h. Thereafter, the cells were harvested by using Trypsin-EDTA solution (Sigma, T4049) and collected via centrifugation in 1000 g for 5 minutes. RNA extraction from the harvested cells was performed using RNX plus kit (RN7713C, Sina clon, IRAN). Briefly, 1 ml ice cold RNXTM –PLUS solution was added to the harvested cells in 2ml microtubes. Samples were vortexed for 10 seconds and incubated 10 minutes at room temperature (RN7713C, Sina clon, IRAN). 200μl chloroform was added to the samples and resuspended, the samples were then incubated on ice for 5 min. Samples were centrifuged at 12000 rpm at 4 ˚C for 15 min. The aqueous phase was transferred to new RNase-free 1.5 ml tube, and an equal volume of Isopropanol was added to the solution, gently mixed and incubated on ice for 15 min. The mixture was centrifuged at 12000 rpm at 4 ˚C for 15 min. Supernatant was discarded and 1 ml of 75% Ethanol was added to the mix, briefly vortexed to dislodge the pellet and then centrifuged at 4 ˚C for 8 min at 7500 rpm. The supernatant was discarded and the pellet was allowed to dry at room temperature for a few minutes. Pellet was dissolved in 30 μl of DEPC treated water. To help dissolve the pellet, the tube was placed in a 55-60 ˚C water bath for 10 min. A NanoDrop 2000c spectrophotometer was employed for concentration and OD measurements. Samples with acceptable OD 260/280 and 260/230 values (~1.8 - 2) were subjected to cDNA synthesis.

The single stranded cDNA was synthesized by using cDNA synthesis kit (K-2261-6, Bioneer, Korea) according to manufacturer's instructions. Briefly, 5μg of RNA was added to cDNA synthesis tube in a final volume of 20 μl DEPC-treated water. The cDNA synthesis tube was placed in a 60 ˚C water bath for 1 h and finally, it was placed in a 95 ˚water bath for 5 min.

qRT-PCR was performed by using the SYBR Green master mix real-time PCR kit (75675 500 RXN ebioscience, USA) according to the manufacturer's instructions. Briefly, 7 μl of SYBR Green Master Mix PCR, 0.35 μl forward and reverse primers from a 4μmol stoke, 0.7 μl of diluted cDNA template and 5.95 μl of DEPC treated water were added to tube. qRT-PCR was done as follows: initial denaturation at 95 °C for 3 min, 40 cycles of denaturation at 95 °C for 15 sec, annealing at 60 °C for 60 sec and elongation at 72 °C for 5 min. The GAPDH (endogenous housekeeping gene) gene was used as an internal control. Quantitative real-time PCR was performed with Rotor-Gene 6000(version: 1.7) to determine CT values and the threshold was adjusted to 0.1 (inside the exponential phase). Delta CT values were calculated in relation to GAPDH CT values by the 2^-Ct^ method, in which ΔCt represents the difference between the CT value of target genes and the CT value of GAPDH(46). 

**Statistical analysis**

Each experiment was carried out in triplicate. All data are expressed as means ± SD. One-way analysis of variance (ANOVA) was performed with the Dennett’s test, using software Graph Pad Prism 6. Significant differences were shown by “*”, “**”, “***” and “****” respectively for 0.05, 0.01, 0.001 and 0.0001significance levels.

## Results

***Streptomyces Levis ABRIINW111*** **killed SW480 colon cancer cells.**

**Figure 1:**
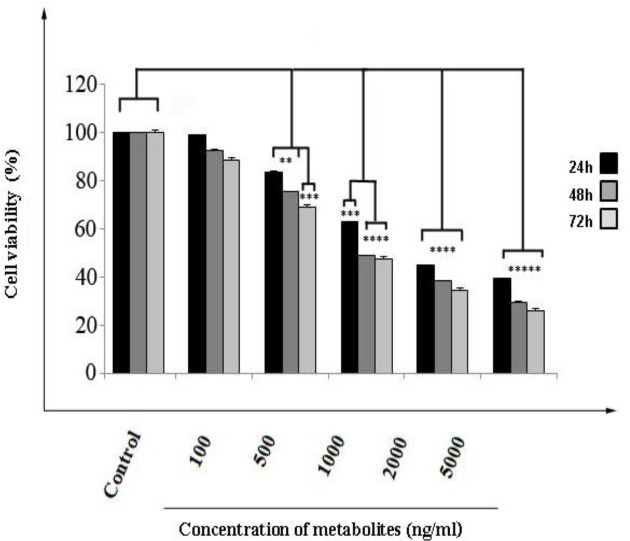
Cell cytotoxicity was examined by MTT assay. SW480 cells were incubated with indicated concentrations of *Streptomyces levis* metabolites for 24, 48 and 72 h. Cell growth was proportional to absorbance at a wave length of 570-630 nm. Values are expressed relative to the control and as mean ± SD of three independent experiments (P < 0.001

MTT assay was performed for evaluating the cytotoxicity and cell viability of SW480 colon cancer following incubation with *Streptomyces Levis ABRIINW111*. Metabolites inhibited cell growth and reduced viability based on the dose and time dependency. Cells were treated for 24, 48 and 72 h use in final concentration of 100, 500, 1000, 2000, 5000 ng/ml metabolites. Viability decreased significantly to 63.27, 48.95 and 47.58 in 1000 ng/ml after 24, 48 and 72 h respectfully. Concentration of 1000 ng/ml was chosen as IC50 value (Figure 1).

***Streptomyces Levis ABRIINW111 altered*** **genes expression in colon cancer cells.**

**Figure 2:**
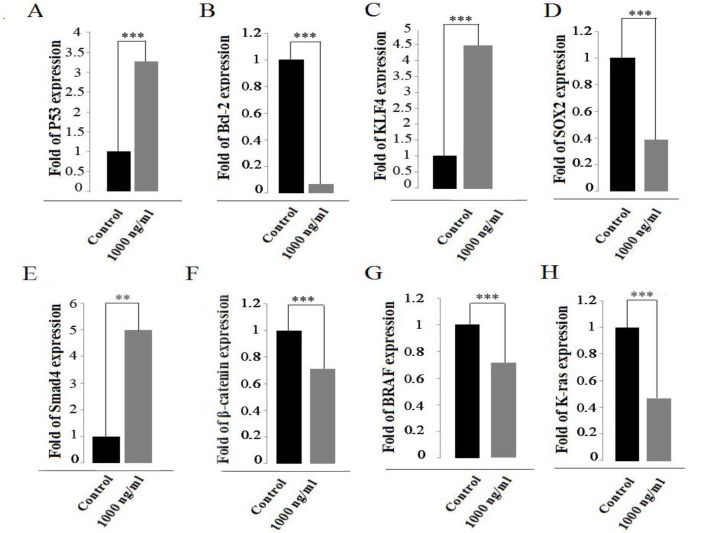
Effect of *S. levis* ABRIINW111 secondary metabolites on gene expression of p53 (***P<0.0001) (A), Bcl-2(***P<0.0001) (B), KLF4(***P<0.0001) (C), SOX2 (***P<0.0001) (D) SMAD4(**P<0.001) (E), β-catenin (***P<0.0001) (F), BRAF (***P<0.001) (G), K-ras (***P<0.0001) (H) gene expression

We treated SW480 cells with metabolites using 1000 ng/ml in final concentration for 48 h. qRT-PCR was performed for harvested cells to evaluate Bcl-2, P53, SOX2, KLF4, β-Catenin, SMAD4, K-ras, BRAF fold expression. P53 gene expression – pro-apoptotic gene involved in apoptosis and cell cycle- increased 3-fold in treated cells (Figure 2A). Also, BCl-2 gene expression – anti-apoptotic gene- was significantly decreased to 0.1 fold of expression in treated cells (Figure 2B).

KLF4 – tumor suppressor - gene expression was significantly increased to 4.5 fold of expression in treated cells (Figure 2C) and also, SOX2 gene expression- oncogenic gene - was significantly decreased to 0.4 fold of expression in treated cells(Figure 2D). 

SMAD4 gene expression - tumor suppressor- was significantly increased 5 fold of expression in treated cells (Figure2E) and β-catenin gene expression – proto oncogene gene - was significantly decreased to 0.6 fold of expression in treated cells (Figure 2F).

BRAF gene expression – proto-oncogene -was significantly decreased to 0.7 fold of expression in treated cells (Figure2G) and K-ras gene expression – the most common mutated gene in ras family- was significantly decreased to 0.4 fold of expression in treated cells (Figure 2H).

## Discussion

Actinomycets, especially Streptomyces sp., the most important source for bioactive compounds are gram positive bacteria found in fresh water, plants surface, marine and terrestrial environments. The exploration of new bioactive compounds has led to the discovery of a new strain which can produce novel useful bioactive compounds (38, 47, 48). In this study, we focused on anti-cancer activity of diethyl ether extracted compounds of *Streptomyces Levis* ABRIINW111 on colon cancer. We showed that diethyl ether extracted compounds have an effect on Bcl-2, P53, SOX2, KLF4, β-catenin, Smad4, K-ras and BRAF genes expression. 

P53 is a tumor suppressor and by controlling cell cycle progression, apoptosis and by inhibiting angiogenesis is able to maintain genomic stability. Also, studies revealed that the Bcl-2 family control the apoptosis by activation of Bax or inhibition of Bcl-2. P53 expression can inhibit Bcl-2 and Bcl-XL expression (13, 49-51). Our result showed that over expression of P53 in treated colon cancer cells with extracted metabolites could downregulate Bcl-2 as an anti-apoptotic, so it could induce apoptosis in P53 dependent pathways. 

SOX2 as a member of the SOX gene family is expressed in human colon cancer. High expression of SOX2 is correlated with a poor prognosis, relapse, and lower survival of patients with colon cancer (52, 53). In the other hand, studies reported that Klf4 as a tumor suppressor plays key roles during the differentiation, proliferation and apoptosis (54-59). There is strong evidence that in colonic adenomas and carcinomas reduction of protein and mRNA level of Klf4 is observed in comparison with normal colonic tissues (60). Our result showed that after 48 h, extracted metabolites could decrease the expression of SOX2, whereas the fold expression of KLF4 was increased.

All of the tumors exhibited increased β-catenin protein compared with normal tissues. It was demonstrated that with the stimulation of epithelial cells through epidermal growth factor (EGF), β- and γ-catenin become tyrosine-phosphorylated. Additionally, a direct association of β-catenin with the EGF-receptor (EGF-R) was shown in vitro (61).

TGF-p signaling pathway transits growth inhibitory signals from the cell surface to the nucleus and Smad4/Dpc4 is a key element of TGF-p signaling pathway. Mutations in SMAD4 have been reported in human pancreatic and colorectal tumors (27, 28, 62). In this study, after 48 h treatment, fold expression of β-catenin was decreased and fold expression of SMAD4 was increased, significantly. 

K-ras, a member of the RAS family of genes, is one of the most noticeable proto-oncogenes in colon cancer. The activated K-ras activates BRAF as a primary downstream target protein. BRAF, serine- threonine protein kinase, acts as a mediator of the K-ras signal toward the downstream effectors such as Mitogen- activated protein (MAP) to increase cell proliferation. Thus, alteration of K-ras seems to promote colon-cancer formation (63, 64).

 Here we showed that K-ras and BRAF fold expression were decreased in colon cancer cells treated with extracted metabolites. These findings show that the crude extracted metabolites have anti-proliferative activity and can inhibit cancer cells proliferation. 

In summary, we have demonstrated that diethyl ether extracted metabolites of *Streptomyces Levis* ABRIINW111 have anti-carcinogenic effects on colon cancer and can alter anti-apoptotic, pro-apoptotic and oncogenes genes expression in treated cells. Also, extracted metabolites as natural products can be a good choice for cancer therapy but more studies are required to characterize the exact structure of metabolites and validate the clinical significance of our findings.

## References

[1] Christopoulou A (2004). Chemotherapy in metastatic colorectal cancer. Tech Coloproctol.

[2] Arber N, B Levin ( 2005). Chemoprevention of colorectal cancer: ready for routine use?. Recent Results Cancer Res.

[3] Rodriguez M, Du GJ, Wang CZ, Yuan CS (2010). Letter to the editor: Panaxadiol's anticancer activity is enhanced by epicatechin. Am J Chin Med.

[4] Fearon ER, Vogelstein A (1990). genetic model for colorectal tumorigenesis. Cell.

[5] Vogelstein B, Kinzler KW (2002). The genetic basis of human cancer.

[6] Danial NN, Korsmeyer SJ (2004). Cell death: critical control points. Cell.

[7] Cory S, Adams JM (2002). The Bcl2 family: regulators of the cellular life-or-death switch. Nat Rev Cancer.

[8] Jeong SY, Seol DW (2008). The role of mitochondria in apoptosis. BMB Rep.

[9] Adams JM, Cory S (1998). The Bcl-2 protein family: arbiters of cell survival. Science.

[10] Thees S, Hubbard GB, Winckler J, Schultz C, Rami A (2005). Specific alteration of the Bax/Bcl2 ratio and cytochrome c without execution of apoptosis in the hippocampus of aged baboons. Restor Neurol Neurosci.

[11] Fernandes-Alnemri T, Litwack G, Alnemri ES (1994). CPP32, a novel human apoptotic protein with homology to Caenorhabditis elegans cell death protein Ced-3 and mammalian interleukin-1 beta-converting enzyme. J Biol Chem.

[12] Oliver FJ, Rubia G De la, Rolli V, Ruiz-Ruiz MC, Murcia G De, Me´nissier-De Murcia J (1998). Importance of poly (ADP-ribose) polymerase and its cleavage in apoptosis Lesson from an uncleavable mutant. J Biol Chem.

[13] Somasundaram K (2000). Tumor suppressor p53: regulation and function. Front Biosci.

[14] Oda E, Ohki R, Murasawa H, Nemoto J, Shibue T, Yamashita T (2000). Noxa, a BH3-only member of the Bcl-2 family and candidate mediator of p53-induced apoptosis. Science.

[15] Toshiyuki M, Reed JC (1995). Tumor suppressor p53 is a direct transcriptional activator of the human bax gene. Cell.

[16] Nakano K, Vousden KH (2001). PUMA, a novel proapoptotic gene, is induced by p53. Mol Cell.

[17] Kamachi Y, Uchikawa M, Kondoh H (2000). Pairing SOX off: with partners in the regulation of embryonic development. Trends Genet.

[18] Shields JM, Christy RJ, Yang VW (1996). Identification and characterization of a gene encoding a gut-enriched Krüppel-like factor expressed during growth arrest. J Biol Chem.

[19] Segre JA, Bauer C, Fuchs E (1999). Klf4 is a transcription factor required for establishing the barrier function of the skin. Nat Genet.

[20] Yet SF, McA'Nulty MM, Folta SC, Yen HW, Yoshizumi M, Hsieh CM (1998). Human EZF, a Krüppel-like zinc finger protein, is expressed in vascular endothelial cells and contains transcriptional activation and repression domains. J Biol Chem.

[21] Ohnishi S, Ohnami S, Laub F, Aoki K, Suzuki K, Kanai Y (2003). Downregulation and growth inhibitory effect of epithelial-type Krüppel-like transcription factor KLF4, but not KLF5, in bladder cancer. Biochem Biophys Res Commun.

[22] Zhang W, Geiman DE, Shields JM, Dang DT, Mahatan CS, Kaestner KH (2000). The gut-enriched Krüppel-like factor (Krüppel-like factor 4) mediates the transactivating effect of p53 on the p21 WAF1/Cip1 promoter. J Biol Chem.

[23] Chen X, Johns DC, Geiman DE, Marban E, Dang DT, Hamlin G (2001). Krüppel-like factor 4 (gut-enriched Krüppel-like factor) inhibits cell proliferation by blocking G1/S progression of the cell cycle. J Biol Chem.

[24] Aberle H, Butz S, Stappert J, Weissig H, Kemler R, Hoschuetzky H (1994). Assembly of the cadherin-catenin complex in vitro with recombinant proteins. J Cell Sci.

[25] Hülsken J, Birchmeier W, Behrens J (1994). E-cadherin and APC compete for the interaction with beta-catenin and the cytoskeleton. J Cell Biol.

[26] Moon RT, Brown JD, Yang-Snyder JA, Miller JR (1997). Structurally related receptors and antagonists compete for secreted Wnt ligands. Cell.

[27] Heldin CH, Miyazono K, Ten Dijke P (1997). TGF-beta signalling from cell membrane to nucleus through SMAD proteins. Nature.

[28] Schmierer B, Hill CS (2007). TGF (beta)-SMAD signal transduction: molecular specificity and functional flexibility. Nat Rev Mol Cell Biol.

[29] Andreyev HJ, Norman AR, Cunningham D, Oates JR, Clarke PA (1998). Kirsten ras mutations in patients with colorectal cancer: the multicenter “RASCAL” study. J Natl Cancer Inst.

[30] Davies H, Bignell GR, Cox C, Stephens P, Edkins S, Clegg S (2002). Mutations of the BRAF gene in human cancer. Nature.

[31] Arber N, Shapira I, Ratan J, Stern B, Hibshoosh H, Moshkowitz M (2000). Activation of cK-ras mutations in human gastrointestinal tumors. Gastroenterology.

[32] Bos JL, Fearon ER, Hamilton SR, Verlaan-De Vries M, Van Boom JH, Van Der Eb AJ (1987). Prevalence of ras gene mutations in human colorectal cancers. Nature.

[33] Arber N, Levin B (2005). Chemoprevention of colorectal cancer: ready for routine use?. Curr Top Med Chem.

[34] Capon DJ, Seeburg PH, McGrath JP, Hayflick JS, Edman U, Levinson AD (1982). Activation of Ki-ras2 gene in human colon and lung carcinomas by two different point mutations. Nature.

[35] Nigro JM, Baker SJ, Preisinger AC, Jessup JM, Hostetter R, Cleary K (1989). Mutations in the p53 gene occur in diverse human tumour types. Nature.

[36] Stallmach A, Zeitz M (1991). Identification of a chromosome 18q gene that is altered in colorectal cancers. Z Gastroenterol.

[37] Fiedler HP, Bruntner C, Bull AT, Ward AC, Goodfellow M, Potterat O (2005). Marine actinomycetes as a source of novel secondary metabolites. Antonie Van Leeuwenhoek.

[38] Huang S, Houghton PJ (2001). Resistance to rapamycin: a novel anticancer drug. Cancer Metastasis Rev.

[39] Vezina C, A Kudelski, S Sehgal (1975). Rapamycin (AY-22,989), a new antifungal antibiotic Taxonomy of the producing streptomycete and isolation of the active principle. J Antibiot.

[40] Sehgal S, Baker H, Vézina C (1975). Rapamycin (AY-22,989), a new antifungal antibiotic Fermentation isolation and characterization. J Antibiot.

[41] Pai MR, Acharya LD, Udupa N (2004). Evaluation of antiplaque activity of Azadirachta indica leaf extract gel—a 6-week clinical study. J Ethnopharmacol.

[42] Surh Y (1999). Molecular mechanisms of chemopreventive effects of selected dietary and medicinal phenolic substances. Mutat Res.

[43] Sadigh-Eteghad S, Dehnad A, Shanebandi D, Khalili I, Razmarayii N, Namvaran A (2011). Identification and characterization of a Streptomyces sp isolate exhibiting activity against multidrug-resistant coagulase-negative Staphylococci. Vet Res Commun.

[44] Mosavi MA, Dehnad AR, Ghanbarvad F (111). Growth Inhibition and Induction of Apoptosis by Ether Soluble Metabolites of Streptomyces sp. ABRIINW.

[45] Moosavi M, F Ghanbarvad, A Dehnad (2012). Growth inhibition and induction of apoptosis by ether soluble metabolites of Streptomyces sp abriinw 111 in human myeloid leukemia k562 cell line. Cell Tissue Journal.

[46] Livak KJ, Schmittgen TD (2001). Analysis of relative gene expression data using real-time quantitative PCR and the 2− ΔΔCT method. Methods.

[47] Blunt JW, Copp BR, Keyzers RA, Munro MH, Prinsep MR (2007). Marine natural products. Natural Prod Rep.

[48] Cragg GM, Kingston DG, Newman DJ (2011). Anticancer agents from natural products.

[49] Lane DP (1993). Cancer. A death in the life of p53. Nature.

[50] Dameron KM, Volpert OV, Tainsky MA, Bouck N (1994). Control of angiogenesis in fibroblasts by p53 regulation of thrombospondin-1. Science.

[51] Levine AJ (1997). p53, the cellular gatekeeper for growth and division. Cell.

[52] Saigusa S, Tanaka K, Toiyama Y, Yokoe T, Okugawa Y, Ioue Y (2009). Correlation of CD133, OCT4, and SOX2 in rectal cancer and their association with distant recurrence after chemoradiotherapy. Ann Surg Oncol.

[53] Ong CW, Kim LG, Kong HH, Low LY, Iacopetta B, Soong R (2010). CD133 expression predicts for non-response to chemotherapy in colorectal cancer. Mod Pathol.

[54] Turner J, Crossley M (1999). Mammalian Krüppel-like transcription factors: more than just a pretty finger. Trends Biochem Sci.

[55] Philipsen S, Suske G (1999). A tale of three fingers: the family of mammalian Sp/XKLF transcription factors. Nucleic Acids Res.

[56] Dang DT, Pevsner J, Yang VW (2000). The biology of the mammalian Krüppel-like family of transcription factors. Int J Biochem Cell Biol.

[57] Black AR, Black JD, Azizkhan‐Clifford J (2001). Sp1 and krüppel‐like factor family of transcription factors in cell growth regulation and cancer. J Cell Physiol.

[58] Kaczynski J, Cook T, Urrutia R (2003). Sp1-and Krüppel-like transcription factors. Genome Biol.

[59] Suske G, Bruford E, Philipsen S (2005). Mammalian SP/KLF transcription factors: bring in the family. Genomics.

[60] Dang DT, Bachman KE, Mahatan CS, Dang LH, Giardiello FM, Yang VW (2000). Decreased expression of the gut‐enriched Krüppel‐like factor gene in intestinal adenomas of multiple intestinal neoplasia mice and in colonic adenomas of familial adenomatous polyposis patients. FEBS Lett.

[61] Hoschuetzky H, Aberle H, Kemler R (1994). Beta-catenin mediates the interaction of the cadherin-catenin complex with epidermal growth factor receptor. J Cell Biol.

[62] Massagué J (1998). TGF-β signal transduction.

[63] Barbacid M (1987). Ras genes. Annu Rev Biochem.

[64] Larki P, Gharib E, Taleghani MY, Khorshidi F, Nazemalhosseini-Mojarad E, Asadzadeh Aghdaei H (2017). Coexistence of KRAS and BRAF Mutations in Colorectal Cancer: A case report supporting the concept of tumoral heterogeneity. Cell J.

